# Modelling the transformation of energy-intensive industries based on site-specific investment decisions

**DOI:** 10.1038/s41598-024-78881-7

**Published:** 2024-12-18

**Authors:** Marius Neuwirth, Tobias Fleiter, René Hofmann

**Affiliations:** 1 Fraunhofer Institute for Systems and Innovation Research (ISI), Breslauer Str. 48, 76139 Karlsruhe, Germany; 2 Institute of Energy Systems and Thermodynamics, TU Wien, Getreidemarkt 9, Wien, 1060 Austria

**Keywords:** Modelling, Investment decision, Energy-intensive industry, Innovative technologies, Site-specific, CO_2_-neutral industry, Hydrogen energy, Energy grids and networks, Climate-change mitigation

## Abstract

The transition towards climate-neutral industry is a challenge, particularly for heavy industries like steel and basic chemicals. Existing models for assessing industrial transformation often lack spatial resolution and fail to capture individual investment decisions. Consequently, the spatial interplay between industry transformation, energy availability, infrastructure availability, and the dynamics of discrete investments is inadequately addressed. Here we present a site-specific approach that considers individual industrial sites to simulate discrete investment decisions. The investment decision is modelled as a discrete choice among alternative technologies with their total cost of ownership as the main decision criterion. Process costs depend on the scenario-specific assumptions, such as energy carrier prices, policy instruments and local infrastructures. The age of production units and their reinvestment cycles are considered the main restrictions on the dynamics of the transition. The results provide high spatial resolution to capture the spatial and temporal dynamics of industry transition under varying process and policy assumptions. The presented model and its results can be coupled with energy system models to assess the implications of site-specific industry transition on energy system related research questions. We conduct an exemplary case study for a transformation pathway of the European primary steel production.

**Supplementary Information:**

The online version contains supplementary material available at 10.1038/s41598-024-78881-7.

## Introduction

The industry sector is one of the largest emitting sectors and needs large amounts of fossil energy carriers for energy and feedstock use, especially in heavy industries^1^. Therefore, these industries play a key role in achieving ambitious climate mitigation targets because they are limited by technical restrictions to switch to low-carbon processes. In the future, climate-neutral industries will require large quantities of climate-neutral energy carriers, including electricity, hydrogen, synthetic fuels, and biomass. At the same time, there is still high uncertainty about the technological direction of industry transition and the role of individual energy carriers. Energy system models are used to investigate alternative pathways to inform decision-makers about feasibility and costs. However, as the energy transition progresses, the need for higher resolution becomes increasingly important to accurately capture the complexities and dynamics of industrial transformation and its implications for the energy system.

Thus, understanding of spatial and temporal factors of industry decarbonisation is crucial for developing reliable pathways. The spatial dimension includes the development of the energy demand per energy carrier with georeferenced resolution as well as the consideration and dependence of existing and planned infrastructure.

Temporal factors involve the economic viability of investments and the current industry structure. Economic viability depends on factors like energy carrier prices and investment costs, while current processes, typical reinvestment cycles and the age of individual plants represent the industry structure. Some plants may not have recouped their investment, tying up significant capital and limiting further investments. Additionally, space constraints at many industry sites complicate the construction of new plants. These restrictions on available capital and space make parallel ramp-up efforts challenging. As a consequence, investments into innovative processes are most feasible when the old plant is depreciated and at the end of its life cycle, significantly affecting the temporal ramp-up of new climate-neutral processes.

In general, modelling approaches to investigate energy transition pathways are becoming more detailed to better predict how investments and technological improvements spread in various sectors, such as the electricity market, transport, residential, and industry sectors^2–7^.

However, the industry sector is diverse, with various subsectors, products, and processes. Some subsectors require large-scale investments in new production units, while others can achieve fuel switching with smaller investments in individual processes^8,9^. Thus, different modelling approaches exist for representing the heterogeneous industry sector, ranging from assessments of single processes^10,11^ to model-based scenarios on subsectors^12–14^ or specific products^15–17^. The known instances of industry implementation are often very aggregated due to the integration within complex energy system models (ESMs) and integrated assessment models (IAMs)^18,19^. Examples are the PRIMES model^20^ or frameworks such as TIMES^21–23^ or MUSE^19^, where industries are often reflected by representatives for a stakeholder group. These representatives act based on exogenous assumptions and statistical distributions because the models focus on the mechanisms of entire energy systems rather than on detailed industry sector analysis^24^. Consequently, the respective process diffusion indicates the weighted interests of the representative groups, which does not fit for the entire industry landscape due to its heterogeneity. Most accurate holistic approaches of the entire industry sector so far consider sub-sectoral bottom-up process shares in stock models^25^. Nearly all known industry sector models operate at the national level due to data availability and scope^25,26^. Thus, the spatial distribution cannot be depicted or only estimated retrospectively using different parameters^25^. In summary, existing approaches either set the diffusion of climate-neutral processes exogenously on national level or calculate the diffusion using stock models with strong assumptions on statistical shares^14,25–27^. Consequently, current methods are limited in two aspects. First, without knowledge about the locations the spatial resolution is insufficient to investigate detailed on the linkages between the energy system, the energy infrastructure, and the industry sector. Second, only rough estimates on the diffusion speed can be provided if the current age structure of the individual industrial plants and typical investment cycles are disregarded.

Summarised, research gaps exist in industry sector modelling particularly in addressing the limitations of diffusion of industrial processes and its spatial distribution. To address these challenges in spatial and temporal dynamics, we demonstrate a modelling approach representing individual sites and plants. The model particularly takes into account the georeferenced industrial plant stock of energy-intensive industries and its plant ages in a site-specific bottom-up manner. Our aim is to propose a transparent method based on key parameters such as cost components, energy demand and emissions. Thus, the model code is available as open source in line with this publication and complete industry datasets for primary steel and basic chemicals from Neuwirth et al. [39] are included.

A description about the context and the scope of the model is presented in “Context and scope of the model”. “Model overview” introduces the model, its structure and input data. The “Mathematical formulation” presents the main variables and equations that influence the discrete investment decision. For demonstrating a use case of the model, we briefly describe a short case study for the European primary steel sites in “Case study: transformation of European primary steel production according to the structure of the current plant stock”. A critical “Discussion” is formulated after presenting the exemplary case study, while “Conclusions and outlook” assesses the possibilities of the model and indicates next steps.

## Context and scope of the model

The purpose of this model is to improve the resolution of the industrial sector in the context of energy system analysis. Typically, such sector models are applied in combination with energy system models that cover the broader context of the entire energy system and reflect the market mechanisms for energy markets such as electricity. The models can be applied iteratively by exchanging on energy demand and corresponding prices to imitate the market dynamics. Those market dynamics are covered in ESMs by combining supply effects such as the expansion of renewable capacities, demand sensitivities, and import and export.

Scenario analysis of such individual sector models within the model chains help to provide possible transformation pathways on the demand side, which are mostly aligned with dedicated climate goals. Diffusion pathways for new processes in energy-intensive industries are often set exogenously and nationally according to the given climate goals as data granularity and knowledge about the individual sites within the industry sector is missing.

This modelling approach provides improved spatially highly resolved insights into the structure of the current industries and helps to understand the spatial and timely dynamics for an economic industry transition. In addition to the spatial distribution, the age of the plant stock and the typical investment cycles are the focus of the presented modelling approach when conducting pathways for industrial transformation. Determinants for the investment decision are the availability and existence of energy infrastructure and the economic viability of the considered processes.

The consideration of the mentioned factors are substantiated by the findings of Wesseling et al.^28^. They have examined and characterised the challenges and barriers to the transformation of energy-intensive industries. These industries show high scaling, energy, and capital intensity resulting in long amortisation periods and extended investment cycles, which were identified as critical factors. Prolonged investment cycles offer limited opportunities for technological updates, while low and cyclical profit margins constrain the availability of investment capital. This situation also presents a significant barrier to the successful market entry of new companies, which is a major driver for the diffusion of innovations in other sectors, such as energy and automotive. Additionally, this creates a high dependency on brownfield investments in existing facilities. Besides the restriction from long investment cycles, Wesseling et al.^28^ highlight the availability and accessibility of specific resources and energy carriers as significant constraints for the competitive implementation of innovative processes.

The model does not account for additional constraints commonly found in typical diffusion theories, such as network effects, learning effects, and endogenous market mechanisms, which are particularly relevant for consumer goods. Unlike well-documented learning curves for consumer goods, these learning curves are less evident for large-scale industrial plants, and empirical data is notably scarce, especially for the innovative processes targeted by the presented model. The incorporation of endogenous learning effects (reduction in specific costs with increased cumulative production capacity) is also typically not applicable to just a segment of the entire market, as represented in this approach. A significant portion of the learning is global, thus extending beyond the model’s scope. Nevertheless, some learning effects can be considered through exogenous assumptions regarding efficiency improvements or the reduction of specific investment costs over time.

In summary, the model serves to address various research questions related to industrial transition in the context of energy system analysis. By coupling with energy system models, these results help improve the understanding of the impacts from the interplay between the industrial sector and other parts of the energy system. The following exemplary research questions, which can utilise the improvements and results from this modelling approach, are raised:

1. How is industrial transformation influenced by the age structure, typical reinvestment cycles, and the spatial distribution of industrial plants?
2. How economically competitive are innovative climate-neutral industrial processes, and how do they change under different conditions, including energy prices and policy instruments?
3. What is the spatial development of industrial energy demand and what dependencies arise concerning infrastructures and import strategies?

## Model overview

**Fig. 1:**
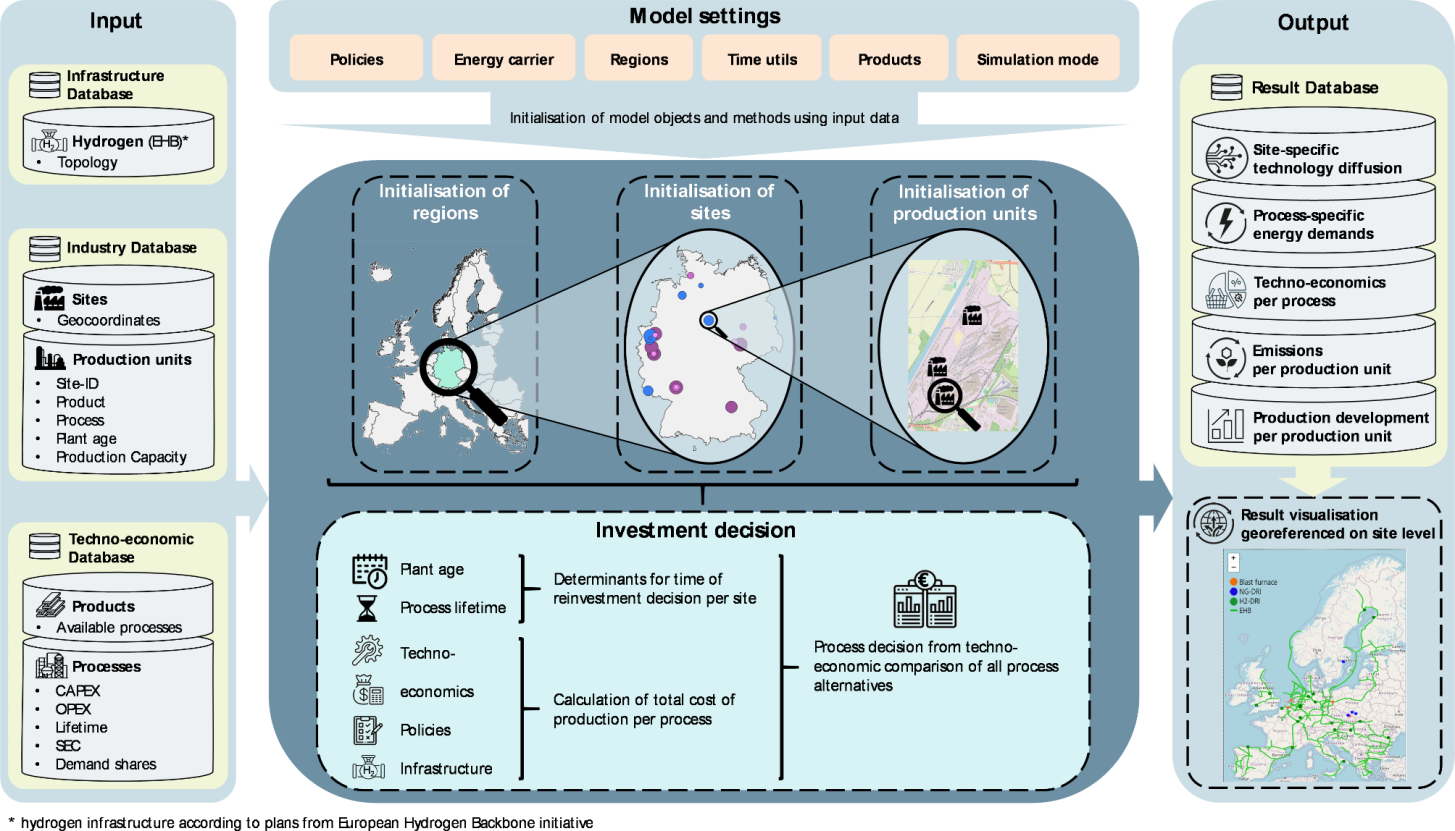
Model overview including required input data, simplified model procedure, and exemplary outputs.

According to the previously introduced “Context and scope of the model”, the approach aims at a high spatial and process resolution by considering individual industry sites and their production units. Each existing industry site is represented with specific information on geolocation, production units and processes as well as corresponding details such as production capacities and emissions. The chosen simulation approach allows to model the transition of the entire fleet of heavy industry production units of selected countries or regions. The investment decisions depend on the scenario-specific assumptions, such as energy carrier prices, policy instruments and local infrastructures. The decision is modelled as a discrete choice among competing processes with their total cost of ownership as the main decision criterion. The age of production units and their reinvestment cycles are considered the main restrictions on the dynamics of the transition. Figure 1 gives an overview over the entire model, including model input output data.

### Software implementation

The model is implemented in Python using commonly known and established packages, listed in the supplementary material. The model classes are defined independently but can be also called by using *mesa*, which is a predefined framework that may act as a scheduler and result visualisation tool in our approach^29^.

The model is based on object-oriented programming because it allows the handling of very data-intensive structures in a clear hierarchy with relatively limited operations. In addition, object-oriented programming complies with the requirements of a flexible framework that gives the opportunity to extend towards agent-based behaviour. The most important indicators that distinguish functional programming from object-oriented programming are compared in the supplementary material. Additionally, thorough reviews and discussions on this topic can be found in the literature^30–33^. We applied the factory pattern for decoupling input data and model initialisation as well as the visitor pattern for collecting and storing model results. The factory pattern is a method commonly known and used for object-oriented models^34,35^. It enables the disconnection of the input databases from the model core and allows considerable flexibility in the creation of objects and fast computation, as the model has no need for database connection during the calculations. With regard to the performance of the model and to avoid the occurrence of unused data, only the information needed for the covered regions out of the scenario settings is imported for each model run. Advantages of the established simulation approach compared to an optimisation lie, i.e. in a better representation of individual investment decisions, the faster computing time and no need for external solvers.

### Model structure

**Fig. 2:**
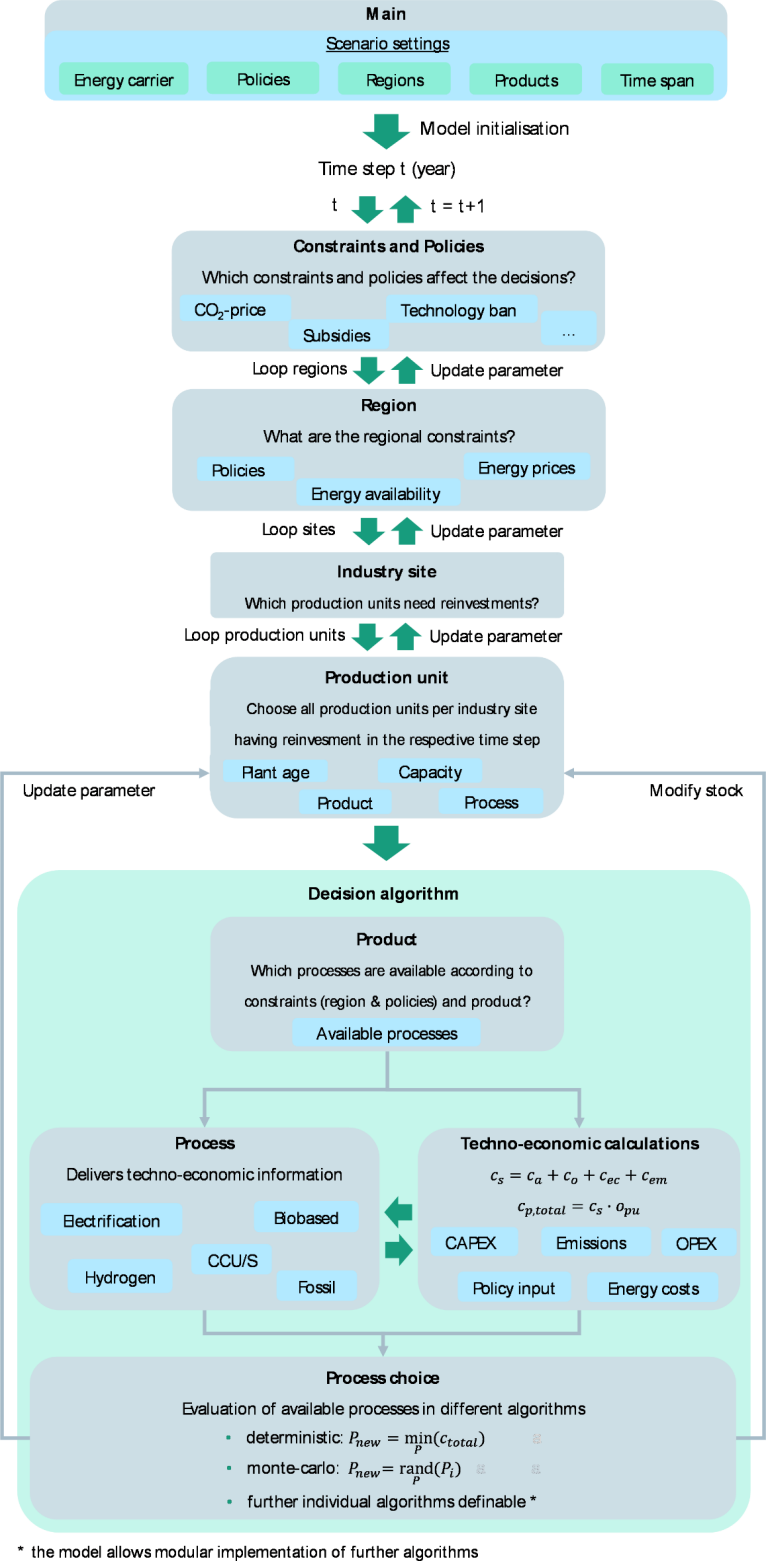
Simple model overview showing the main elements of the model structure and the functional concept of the process decision per industry site in each time step.

Figure 2 shows a simplified model overview and workflow of the model procedure. A global main class defines scenario-specific settings, e.g., modelled regions, products, policies, simulation modes, start years, and simulation time steps. Scenario-specific inputs are energy carrier types and price assumptions as well as policy parameters, which manipulate the main drivers of process choice. The model input and settings can be defined externally for each scenario and model run.

During initialisation, these inputs are assigned by the factory classes to the corresponding level in the model hierarchy because they affect different objects. The simulation steps are discrete time steps performed by the scheduler. These methods are flexible and definable but are typically carried out in yearly increments, as that is the most suitable time step for the use case for which the model is built. At each step, the constraints for each region are updated.

**Fig. 3:**
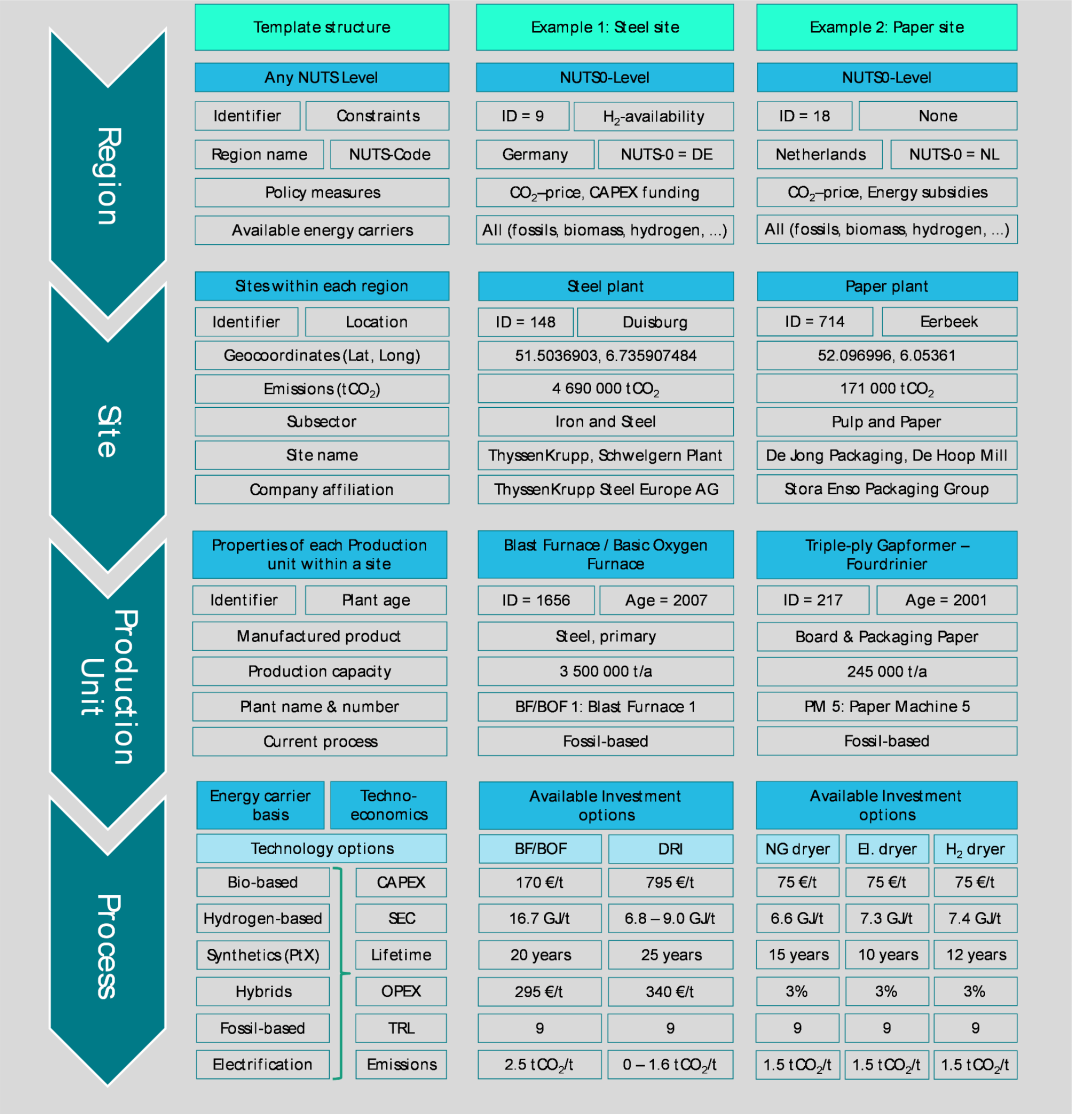
Filled data input according to the “Model structure” for a primary steel production unit and a board and packaging paper production unit.

In general, the implemented approach classifies its objects and methods into four hierarchical levels. The upper level is the region class, which initiates the regions. The model runs through each region per time step. Within the modelled regions, the industrial sites, undergo a decision algorithm. Each site has one or more production units that manufacture specific products using certain processes. Figure 3 shows an example of a completed template structure for existing industrial sites for the given model hierarchy levels.

Next, we briefly present the necessary data assigned to the corresponding level in the hierarchy:

1. *Region*: This class creates all regions defined in the main class. The definition of a region can be flexible. However, it is reasonable to use internationally predefined levels such as the 'Nomenclature of Territorial Units for Statistics’ (NUTS) classification, as this also refers to differences in pricing levels and policy actions. Each region is initialised with a unique identifier (ID). The allocation of the industry sites to the respective region is coupled via this ID. Energy carriers and their associated prices, as time series or parameters, are necessary data inputs for each region. Optional inputs can include any kind of constraint for a region. These may include but are not limited to different types of policy actions, such as process bans, energy availability, subsidies or taxes. Each region contains a defined list of sites.

2. *Site*: Each site is characterised by a unique ID, the subsector to which it refers, geo-coordinates and production units. The input data for an industry site must include the region ID to allocate region-specific parameters. This enables the individual site to act by using information that is given by the subordinate classes for several production units, products and processes. In each time step (usually a year), the model checks whether new investments are required for each site.

3. *Production unit*: Each production unit requires a row of different IDs to connect with. First, site-ID allows mapping to the correct industry site. Knowledge about specific IDs of the manufactured product per production unit enables the connection to the subclass for process-ID options. Further parameters are the yearly production capacity per production unit and the age of the production unit. Based on this, the probability of new investment decisions is calculated, considering the scenario-specific constraints and techno-economic factors at the process level. A production unit can consist of multiple sub-production units, which may produce intermediate products through specific processes. The sub-production units are structured in the same way. One production unit can cover one or multiple industrial plants, depending on the scope of the modelled aggregation. However, given the current data availability, all modelling activities using this model have focused on integrated production plants without sub-production units. Thus, a plant in the following represents a production unit.

4. *Process*: The process class represents the lowest level in the hierarchy of the core model and the initialisation of the industry sites. At the same time, most of the primary data are processed in that part of the model. Here, all specific techno-economic parameters per process identified as alternatives for the production of a specific product are calculated. Most input parameters are needed at the process level. Apart from the product-ID for mapping within the model, time series for CAPEX, OPEX, energy demands, fuel shares and lifetime and emission factors are mandatory.

In the “Mathematical formulation” the rules for the resulting process decision are described. Following this approach, the decision algorithm runs at the site level and is influenced by external constraints (e.g., per region) and site-specific properties. Actions per industry site are carried out if a decision is due whether to reinvest into one of its production units and the corresponding process in the respective year. The basis for site-specific reinvestments is the age of the processes within the respective production units as well as a techno-economic comparison of alternative processes per production unit. The process-specific variables are returned through the hierarchy for processing the techno-economic data in evaluating individual decisions. These site-specific investment decisions based on the age of the individual production units and process-specific techno-economics lead to insights into process diffusion, as the aggregated diffusion dynamics can be explained by individual technology adoption decisions. Variations of the scenario-specific definitions and the exogenous assumptions, such as energy price projections, and the corresponding differences in the results and industrial process decisions give insights into the effects and dependencies. The *mesa* framework supports the applicability of visualisation tools and result creation in combination with the visitor pattern.

### Model input data

**Fig. 4:**
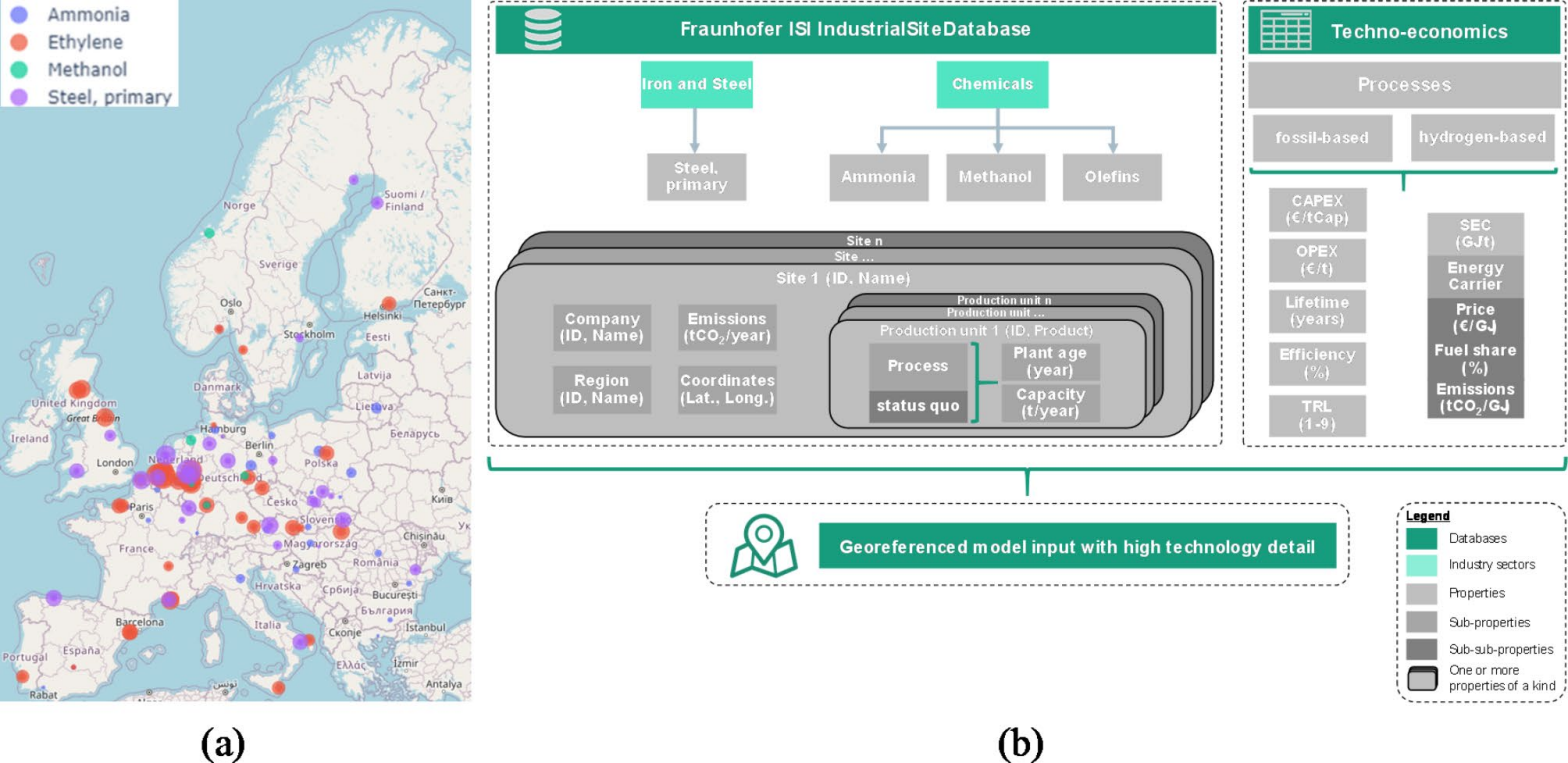
Matching of the Fraunhofer ISI IndustrialSiteDatabase and techno-economic process data.

The model input data are set up as an SQL database and administered by the model via a data interface that connects to the upstream factory classes of each model class. The “Model structure” indicates the need for certain input data, which currently consists of two sources:

First, the Fraunhofer ISI IndustrialSiteDatabase, which includes site-level data for European energy-intensive industries and their main products. The iron and steel, basic chemicals, non-metallic minerals, non-ferrous metals and pulp and paper industry branches are covered with high detail^36–38^. We provide the industry site information for the European primary steel and basic chemical sites, as visualised in Fig. 4a. Definitions of industrial subsectors, products and processes are predetermined by this data structure. The IndustrialSiteDatabase provides data for the individual industry sites with corresponding production units and their products, production capacity, reported EU-ETS emission, plant age and geo-coordinates.

Second, the techno-economic inputs shown in Table 1 are needed for quantifying the different objects in the model structure. In particular, processes need to be described by adding costs such as CAPEX and OPEX, specific energy consumption (SEC) with its type and use and assumptions on future efficiency improvements. For the process-specific techno-economic data, we build on previous work that already address this type of data and extend them with values from recently published literature^25,38^. Scenario-specific inputs are mainly represented by energy carriers with corresponding price assumptions, emission factors and availability per region. Economics for policies such as CO_2_ pricing, funding, subsidies or taxes are also defined in the scenario-specific input. The impact of policies, except from process bans, result in price modifications that influence investment decisions through “External cost-influencing factors”.

**Table 1:** Necessary model inputs used for calculating investment decisions.

Parameter	Description
Process-specific techno-economics	
CAPEX	Capital expenditure (CAPEX) per process in €/t
OPEX	Operational (OPEX) expenditure per process in €/t
SEC	Specific energy consumption (SEC) per energy carrier and type of use in €/GJ
Energetic use	Energy demand for energetic purposes within the process, e.g. direct process heat
Feedstock use	Demand on energy carrier for non-energetic purposes, e.g. fossils in chemical products
Steam generation	Energy demand for steam generation within processes, e.g. drying of paper lanes
Efficiency improvement	Assumed efficiency improvement over time of the respective process in %
Scenario-specific techno-economics	
Energy carrier	Definition of energy carriers used in the modelled industries and processes
Price(-developments)	Price time series per energy carrier in €/GJ
Emission factors	Emission factors per energy carrier in tCO_2_/GJ
Availabilities	Energy availability per region in GJ/a
Policies	Policies manipulate investment decisions by setting constraints at different levels
Process bans	Ban for avoiding investments in specific processes per country
CO_2_ price	Price(-development) for penalty on emitted CO_2_ in €/tCO_2_
Subsidies	Subsidies on single energy carriers per country in €/GJ
Taxes	Taxes on single energy carriers per country in €/GJ
Investment funding	Funding on investment for specific processes or projects per country

These two data sources are automatically matched in a pre-processing step to establish an extensive database dependent on the exogenously defined model settings as input. The data are used to initialise the model according to the template structure of Fig. 3, reflecting the introduced model hierarchy. The required input parameters are connected using the unique identifiers (IDs) of the different levels to initialise the industry sites. Examples 1 and 2 show the detailed granularity of the input data for the respective hierarchy level and model class. The generality of the template structure allows the implementation of very different industries in the same way and simplifies the heterogeneity between them.

A dataset for modelling the entire European primary steel and basic chemical production is published for open source in line with the publication of Neuwirth et al.^39^. We provide these data within the repository of this established approach of the model. Thus, scenarios for the entire European primary steel production and basic chemical industry can be directly applied and modelled.

Figure 4 shows the number of sites and the spatial distribution of the provided data (a) as well as the most important parameters from both sources (b).

Based on the model framework, the whole structure and required input data can be adapted for any other sector or use case. However, the model was established to focus on the industry sector, and the structure of the input templates is well suited for this application.

### Model output and visualisation

The aim is to obtain an understanding of the market diffusion of climate-neutral industry processes with a georeferenced site-specific resolution. Accordingly, the model calculates the process-specific energy demands per energy carrier as well as the process- and energy-related emissions per production unit. In addition, the cost components per process, along with the underlying assumptions, are provided as results for analysing the techno-economic indicators. To collect the model results, the so-called visitor pattern is used. Similar to factory patterns, the main advantage is that they work without changing the classes of the objects on which they operate for collecting results to be written. More detailed information on factory and visitor patterns is given in the literature^34,35,40^. For reproducibility, every scenario run is saved in an extra output database. The visualisation of the scenario results is automated by opening a new tab using the python package *mesa-geo*^41^, an add-on of the agent-based modelling (ABM) framework *mesa.* This allows us to directly set up dynamic visualisations and simulate the model scenarios on a georeferenced map. In addition, variables and dynamic figures for selected variables reflecting the stage of the simulation can be implemented.

## Mathematical formulation

Introduced in “Context and scope of the model”, process diffusion in industry faces a variety of sector-specific challenges, such as long investment cycles and high barriers to market entry because of its scale, energy and capital intensity^28^. Moreover, existing economics-based theories and statistical approaches for innovation diffusion have a limited fit to the industry structure, as the restricting parameters differ from those of other sectors. Thus, investment decisions in the energy-intensive industries are more determined by plant age and reinvestment cycles as well as long-term techno-economic driven aspects.

The reinvestment decision is modelled in a discrete manner by simulating the investments of each production unit when it reaches the end of its life in a specific year. As an example, a primary steel manufacturer invests into a relining of its existing blast furnace or into an alternative, such as a direct reduction plant, when the existing blast furnace reaches the end of its lifetime. Regional constraints such as the availability of energy carriers and corresponding prices as well as selected policies affect the decision. Implemented policy options mainly reflect economic aspects and manipulate the decision algorithm through CO_2_ pricing, investment funding, subsidies or taxes on energy carriers or by defining process bans. Considering these scenario-specific regional settings, the model calculates the techno-economics for all available processes. Depending on the regional constraints and the respective policies, processes using direct electrification, hydrogen, biomass, carbon capture and utilisation or storage (CCUS) or fossil energy can compete.

The algorithm for the investment decision then chooses the most economic (cheapest) process available for the respective product (Eq. (1)):

1 $$\:{Inv}_{p}={\text{m}\text{i}\text{n}(c}_{p,total})$$

- $\:{Inv}_{p}$ process evaluated for new investment for the individual production unit
- $\:{c}_{p,total}$ total costs per process

The total production cost for each available process (Eq. (2)) recognised for a probable reinvestment within a production unit is derived from a row of cost calculations (Eqs. (3)–(6)), resulting in the specified total cost and the production output per year.

2 $$\:{c}_{p,total}={c}_{s}\cdot\:{o}_{pu}$$

- $\:{c}_{p,total}$ total production cost per process
- $\:{o}_{pu}$ output/production volume per production unit.

### Process-related techno-economics

The basis for the process choice is the total cost of ownership. All calculations are carried out per time step, which is currently implemented as yearly steps. Therefore, the total specific costs per ton of product (Eq. (3)) are calculated as the sum of specific annual capacity costs, specific operational costs and costs for energy carrier use. External influences such as different policy-related aspects or infrastructure access are covered and described in “External cost-influencing factors”:

3 $$\:{c}_{s}={c}_{a}+{c}_{o}+{c}_{ec}+{c}_{ext}$$

- $\:{c}_{s}$ specific total cost per ton of product
- $\:{c}_{a}$ specific annuity of investment per ton of product
- $\:{c}_{o}$ specific operational costs per ton of product
- $\:{c}_{ec}$ specific energy carrier costs per ton of product
- $\:{c}_{ext}$ external influences on specific total cost per ton of product.

Investments per ton of yearly capacity include equipment and supplements needed for the installation of a process and are converted to annual capital costs per ton of product (Eq. (4)) by applying the capital recovery factor and the capacity utilisation factor per year (Eq. (5)).

4 $$\:{c}_{a}={f}_{a}\cdot\:{c}_{i}\cdot\:{f}_{cu}$$

- $\:{f}_{a}$ specific capital recovery factor;
- $\:{c}_{i}$ specific total investments per ton of installed yearly capacity;
- $\:{f}_{cu}$ capacity utilisation factor per year.

5 $$\:{f}_{a}=\frac{{\left(\right(1+r)}^{i})\cdot\:r}{{\left(\right(1+r)}^{i})-1}$$

- *i* depreciation period
- *r* interest rate

The operational costs consider labour, maintenance and non-energetic resources, e.g., iron ore for primary steel production. Each process is defined by its specific energy consumption (SEC) from the input data and related shares of energy carriers that are used to provide the needed energy. The SEC consists of three dimensions: (i) electricity, (ii) fuel, and (iii) feedstock. Using this information multiplied by the corresponding energy carrier prices for the respective years, the energy carrier costs per ton for each process are calculated (Eq. (6)).

6 $$\:{c}_{ec}=\sum\:(SEC\cdot\:{sh}_{ec}\cdot\:{p}_{ec})$$

- $\:SEC$ specific energy consumption per process
- $\:{sh}_{ec}$ share of the specific energy consumption per energy carrier
- $\:{p}_{ec}$ price per energy carrier in the respective year.

### External cost-influencing factors

To calculate variations and analyse the effects of external factors such as policies or infrastructure on the process decision and corresponding diffusion behaviour, different implications for economic calculations are realised. More specifically, carbon pricing, subsidies and energy taxes as well as investment funding and infrastructure (currently: European Hydrogen Backbone (EHB) as future hydrogen infrastructure) access can be recognised and applied by a cost factor (Eq. (7)).

7 $$\:{c}_{ext}={c}_{em}+{c}_{f}+{c}_{sub}+{c}_{tax}+{c}_{inf}$$

- $\:{c}_{em}$ specific emission-related costs from carbon pricing per ton of product
- $\:{c}_{f}$ specific annuity from investment funding per ton of product
- $\:{c}_{sub}$ specific costs from subsidies on energy carrier per ton of product (< 0)
- $\:{c}_{tax}$ specific costs from taxes on energy carrier per ton of product
- $\:{c}_{inf}$ specific annuity from energy infrastructure cost per ton of product

#### Carbon pricing

The European Union’s Emissions Trading System (EU-ETS) is an established instrument intended to reduce greenhouse gas emissions from the participating energy sector and energy-intensive industries^42^. By setting a CO_2_ price, companies in the participating sectors are forced to pay for each tonne of carbon dioxide they emit (Eq. (8)).

8 $$\:{c}_{em}={c}_{em,ec}+{c}_{em,p}$$

- $\:{c}_{em}$ specific emission-related costs per ton of product
- $\:{c}_{em,ec}$ specific emission costs per energy carrier per unit used per ton of product
- $\:{c}_{em,p}$ specific emission costs per process.

We represent the ETS as a simple exogenous time series of a CO_2_ price that is applied for direct (on-site) emissions from energy carrier use (Eq. (9)) and process-related emissions (Eq. (10)).

9 $$\:{c}_{em,ec}={\sum\:{(sh}_{ec}\cdot\:f}_{em,ec}\cdot\:{p}_{CO2})$$

- $\:{f}_{em,ec}$ specific emission factor per unit of used energy carrier; price per ton of emitted CO_2_.
- $\:{p}_{CO2}$ price per ton of emitted CO_2_.

10 $$\:{c}_{em,p}={f}_{em,p}\cdot\:{p}_{CO2}$$

- $\:{f}_{em,p}$ specific process emission factor per ton of product.

Indirect emissions from electricity, biomass and district heating are not considered for the CO_2_-price, as the model is following the definition of the EU-ETS (only scope 1).

#### Funding

Funding is a financial support for capital investments. The implementation is realised in two different ways. On the one hand, the funding of individual projects can be directly dedicated to a single industry site (case 1). On the other hand, an amount of money for investment funding per subsector and product for a fixed time period can be set, and a maximum funding rate per production unit is definable (case 2). Reflecting on the mode of operation, the economics of the process choice are affected by a reduction in the CAPEX, as shown in Eq. (11).

11 $$\:{c}_{if}=\left\{\begin{array}{c}\frac{{c}_{project}}{cap},\:\:case\:1\\\:{c}_{i}\cdot{f}_{f},\:\:case\:2\end{array}\right.$$

- $\:{c}_{if}$ specific investment funding per production unit
- $\:{c}_{project}$ total investments per production unit
- $\:cap$ installed capacity in tons
- $\:{f}_{f}$ maximum funding rate per production unit.

The annuity of investment is thereby affected by the specific investment per ton of installed capacity according to Eq. (12).

12 $$\:{c}_{f}={f}_{a}\cdot\:{c}_{if}\cdot\:{f}_{cu}$$

#### Subsidies

Subsidies represent an option as a policy measure for influencing process choice. In particular, subsidies for renewable energy carriers such as hydrogen may support the economy of climate-neutral processes. A reduction in the energy carrier price increases the attractiveness of processes using the subsided energy carrier. The energy carrier price is eased by the provided political subsidy per unit of energy used in the respective process (Eq. (13)).

13 $$\:{c}_{sub}=\sum\:({SEC}_{p}\cdot\:{sh}_{ec}\cdot\:{s}_{ec})$$

- $\:{s}_{ec}$ subsidy per energy carrier in the respective year.

#### Taxes

Energy carriers become more expensive by adding taxes on top of the energy carrier price. Therefore, processes needing the respective energy carrier are less attractive due to higher production costs (Eq. (14)).

14 $$\:{c}_{tax}=\sum\:({SEC}_{p}\cdot\:{sh}_{ec}\cdot\:{t}_{ec})$$

- $\:{t}_{ec}$ tax per energy carrier in the respective year.

#### Infrastructure

An important aspect for industry when making investment decisions is the availability of energy. Investments in new processes may require alternative energy carriers. Thus, assessing access to the needed amount of the corresponding energy carrier is crucial. To consider these factors and associated costs, existing infrastructures and planned infrastructure projects are fed into the model. As an example for hydrogen, the plans of the EHB initiative serve as an exogenous input to check the possible availability per site by checking the distance to the next pipeline with information about the year of first commissioning for each segment. Based on the calculated distance, a cost factor per distance unit (e.g., kilometre) affects the cost and attractiveness of hydrogen-based processes (Eq. (15)).

15 $$\:{c}_{inf}=d\cdot\:{f}_{inf}$$

- $\:{c}_{inf}$ specific cost per ton of product due to infrastructure costs
- $\:d$ distance between site and next pipeline segment per energy carrier
- $\:{f}_{inf}$ cost factor per kilometre distance to next pipeline.

### Fuel switch

Process choice and investment in processes using new energy carriers entail risks. Thus, flexibility in the use of different energy carriers within restrictive technical limits and hybrid process options is highly attractive. The driver for deciding on the share of used energy carrier per year is the comparison of energy carrier costs seen per industrial site. The total costs are derived from the energy carrier prices, costs for emissions, subsidies and taxes per energy carrier (Eq. (16)).

16 $$\:{c}_{ec,j}={SEC}_{p}\cdot\:({p}_{ec,j}+{t}_{ec,j}-{s}_{ec,j}+{c}_{em,ec,j})$$

- $\:{c}_{ec,j}$ specific total cost per energy carrier j per ton of product
- $\:{c}_{em,ec,j}$ specific emissions cost per energy carrier j.

The share per energy carrier used for the individual fraction of the specific energy consumption can be limited by a minimum and a maximum value per process and can vary between 0 and 1, representing the range from 0 to 100% of covered energy consumption (Eq. (17)).

17 $$\:0\le\:{sh}_{ec,min}\le\:{sh}_{ec,j}\le\:{sh}_{ec,max}\le\:1$$

- $\:{sh}_{ec,j}$ actual share per energy carrier
- $\:{sh}_{ec,min}$ minimum share per energy carrier
- $\:{sh}_{ec,max}$ maximum share per energy carrier.

In the current state of the model implementation, no heuristic for incremental switching between competing energy carriers is defined. Thus, depending on annual costs, either the minimum or maximum values are chosen (Eq. (18)). If the first energy carrier option is more costly than the second energy carrier option, the processes uses the second energy carrier option to its maximum extend and the first to its minimum.

18 $$\:{sh}_{ec,j}=\left\{\begin{array}{c}{sh}_{ec,min},\:\:{c}_{ec,1}>{c}_{ec,2}\\\:{sh}_{ec,max},\:\:{c}_{ec,1}<{c}_{ec,2}\end{array}\right.$$

- $\:{c}_{ec,1}$ first energy carrier option for the respective process
- $\:{c}_{ec,2}$ second energy carrier option for the respective process.

## Case study: transformation of European primary steel production according to the structure of the current plant stock

We apply and demonstrate the model using a case study for the uptake of hydrogen to supply climate-neutral primary steel production according to the discrete diffusion mechanism based on reinvestments due to the age structure of the current stock. The model results show the possible ramp-up of hydrogen demand in the steel sector and its spatial distribution depending on the EHB. In general, the model results can serve as a basis for a more detailed systemic understanding of process diffusion in industry and its integration into the energy system. The results can help to facilitate the development of policy strategies to support a hydrogen economy in Europe.

### Input data and assumptions

We briefly present the current structure of European primary steel production and its key parameters for the conducted case study of the Fraunhofer ISI IndustrialSiteDatabase. Furthermore, the most important scenario input data and corresponding techno-economic assumptions for processes, energy carrier prices and policies are shown and analysed.

#### European primary steel sites

**Fig. 5:**
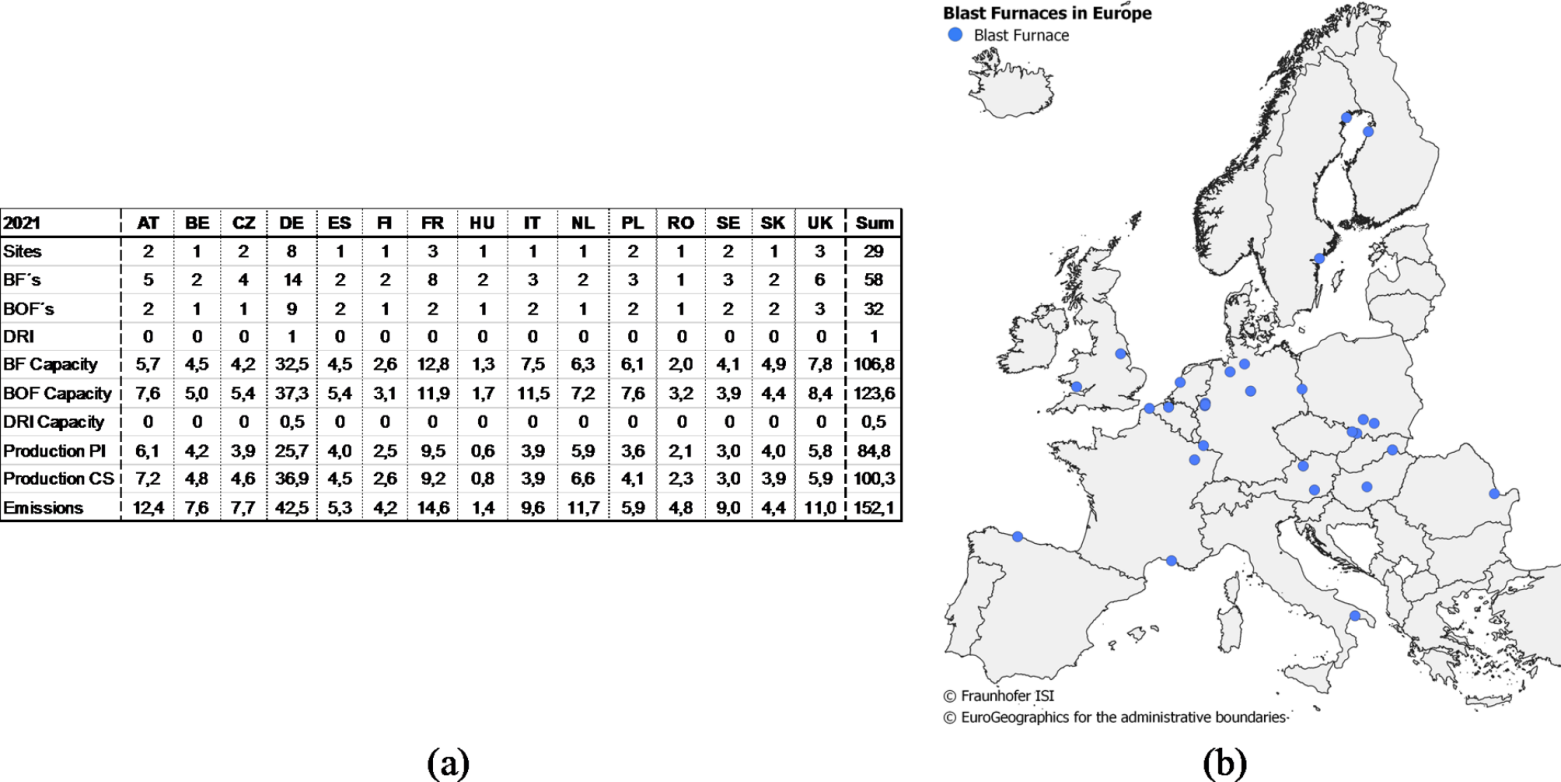
Overview of primary steel sites and their characteristics for 2021 in terms of the number of sites, types of plants, production volumes, and emissions (**a**) and the spatial distribution of European blast furnaces (**b**).

Figure 5a lists the structure of primary steel production per country for 2021 from the IndustrialSiteDatabase presented in “Model input data” and its spatial distribution in Fig. 5b.

In summary, currently, 58 blast furnaces are located at 28 sites in the EU27 + 3, which are dedicated to 15 different countries with a total capacity of approximately 107 Mt of pig iron per year. In total, around 152.1 Mt of greenhouse gas (GHG) emissions are produced per year, and the last refurbishments were conducted from 1971 to 2016.

#### Process and energy assumptions

Current decarbonisation projects and published plans for reducing GHG emissions to reach ambitious climate goals clearly focus on the direct reduction of iron ore^43^. As low carbon investment options in this case study, we allow DRI to be fired by either natural gas or hydrogen, depending on the cost and energy carrier availability.

Table 2 shows the most important parameters and assumptions used for applying the model. Concerning the SEC values, a constant scrap rate of 20% is assumed for all process alternatives^44^.

**Table 2:** Key process parameters used as input data for producing primary steel via BF/BOF or DRI (according to^45^).

Parameter	BF/BOF	DRI/EAF (NG)	DRI/EAF (H_2_)
SEC fuel (GJ/t)	16.7	9.0	6.8
SEC electricity (GJ/t)	1.1	2.1	1.7
Lifetime (years)	20	25	25
CAPEX (€/tCap)	170	795	795
OPEX (€/t)	295	340	340
Main energy carrier	Coal	Natural gas	Hydrogen

**Fig. 6:**
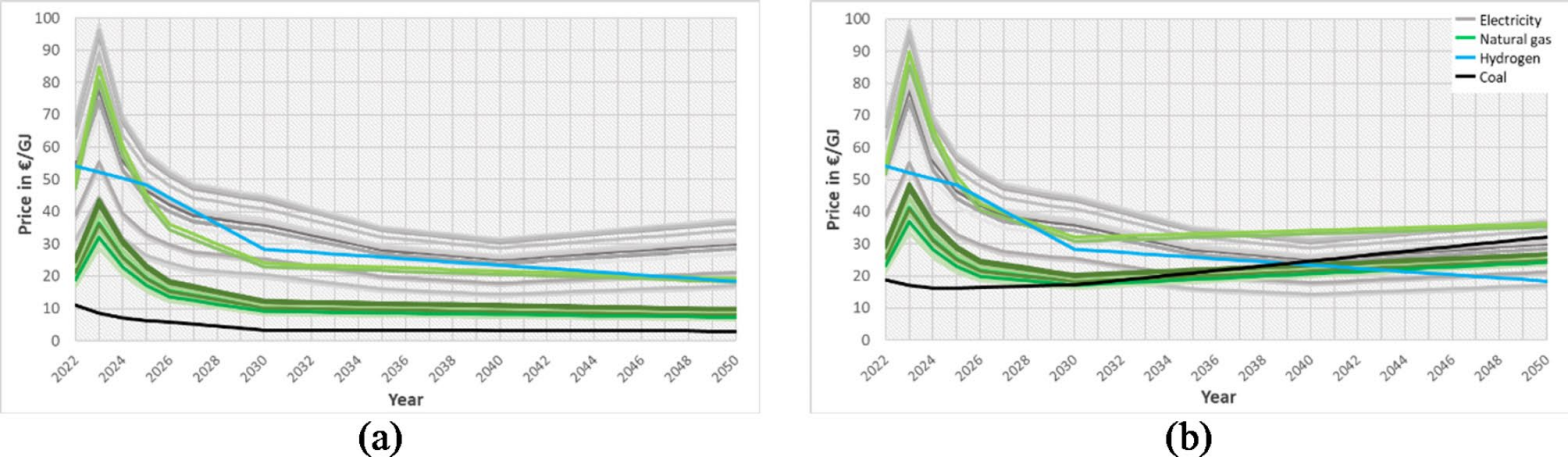
Assumed price developments for the most interesting energy carriers of the case study: electricity, natural gas, hydrogen and coal without carbon pricing (**a**) and with carbon pricing (**b**).

Figure 6a displays the assumed average energy carrier prices per year and corresponding forecasts on the price developments in the countries covered. Without carbon pricing, coal will remain the least expensive, and hydrogen will be the most expensive energy carrier after 2025. The corresponding energy price assumptions for adding recognised carbon pricing are shown in Fig. 6b. Here, the CO_2_ price increases linearly from 80€/t in 2022 to 300€/t in 2050. By increasing the CO_2_ price, natural gas starts challenging the coal price in the first countries from 2028 onwards, and coal will become the most expensive by 2050. In contrast, hydrogen is already attractive in some countries until 2030, reaches the break-even point compared to coal in 2040 and will be the least expensive starting in 2043.

### Case study results

In this case study, we investigate reinvestment behaviour by applying the CO_2_ price as a policy (CASE 1) and an additional process ban on the fossil blast furnace process (CASE 2). Specifically, we conduct a short interpretation of the model results by comparing investments and final energy demand between both cases and show the spatial development and visualisation for CASE 2.

#### Investments

**Fig. 7:**
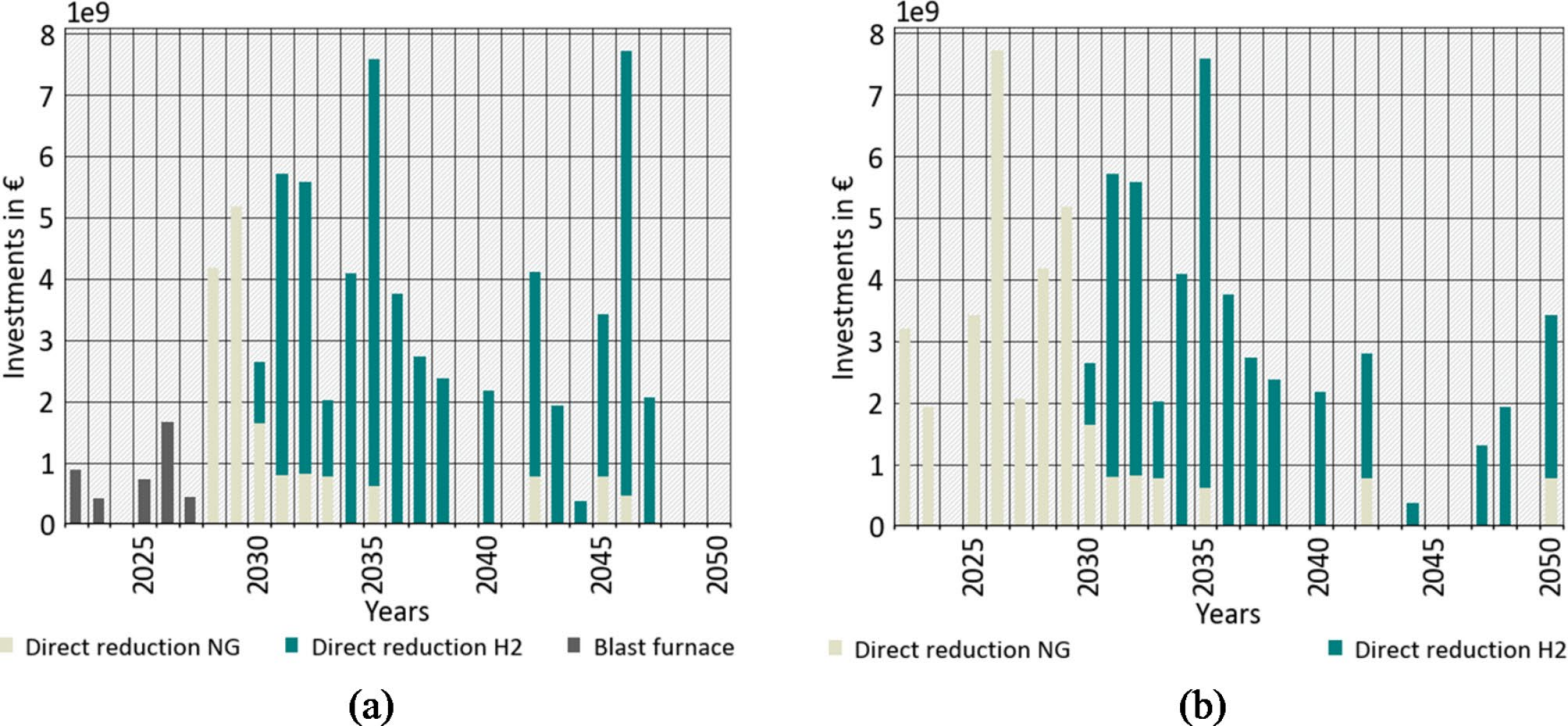
Investment in primary steel sites according to typical reinvestment without a process ban (**a**) and with forbidden reinvestment in blast furnaces (**b**).

Figure 7 shows the resulting reinvestments by applying the assumption from “Input data and assumptions”. For CASE 1 (Fig. 7a), in the early years, blast furnaces remain the most attractive process. In 2028, the first investments into DRI units are made and fired by natural gas. Hydrogen starts to become techno-economically attractive from 2030 onwards. In line with the energy prices in Fig. 6, further DRI investments in the different countries are either planned using natural gas or hydrogen on their first operation. With a theoretical lifetime of 25 years for DRIs, nearly the whole vintage capital stock in Europe will undergo at least one investment decision by 2040. In this case, 17 billion euros will be invested until 2030, of which 9 billion euros will be invested in new blast furnaces. The investment sum until 2040 would result in 52 billion euros of investments. DRI investments from 2045 to 2050 represent the beginning of a second investment phase. In total, 71 billion euros of investments are made by 2050. The cumulative investments are shown in the supplementary material.

CASE 2 (Fig. 7b) realises a process ban for reinvesting in blast furnaces. This reflects the announcements and strategies of European steel manufacturers^43^.

Thus, higher investments of approximately 13 billion euros are necessary in the early years until 2030, as DRI investments are more expensive than BF refurbishments. In summary, the calculated investments are 30 billion euros by 2030, 67 billion euros by 2040 and 77 billion euros by 2050.

#### Final energy demand

**Fig. 8:**
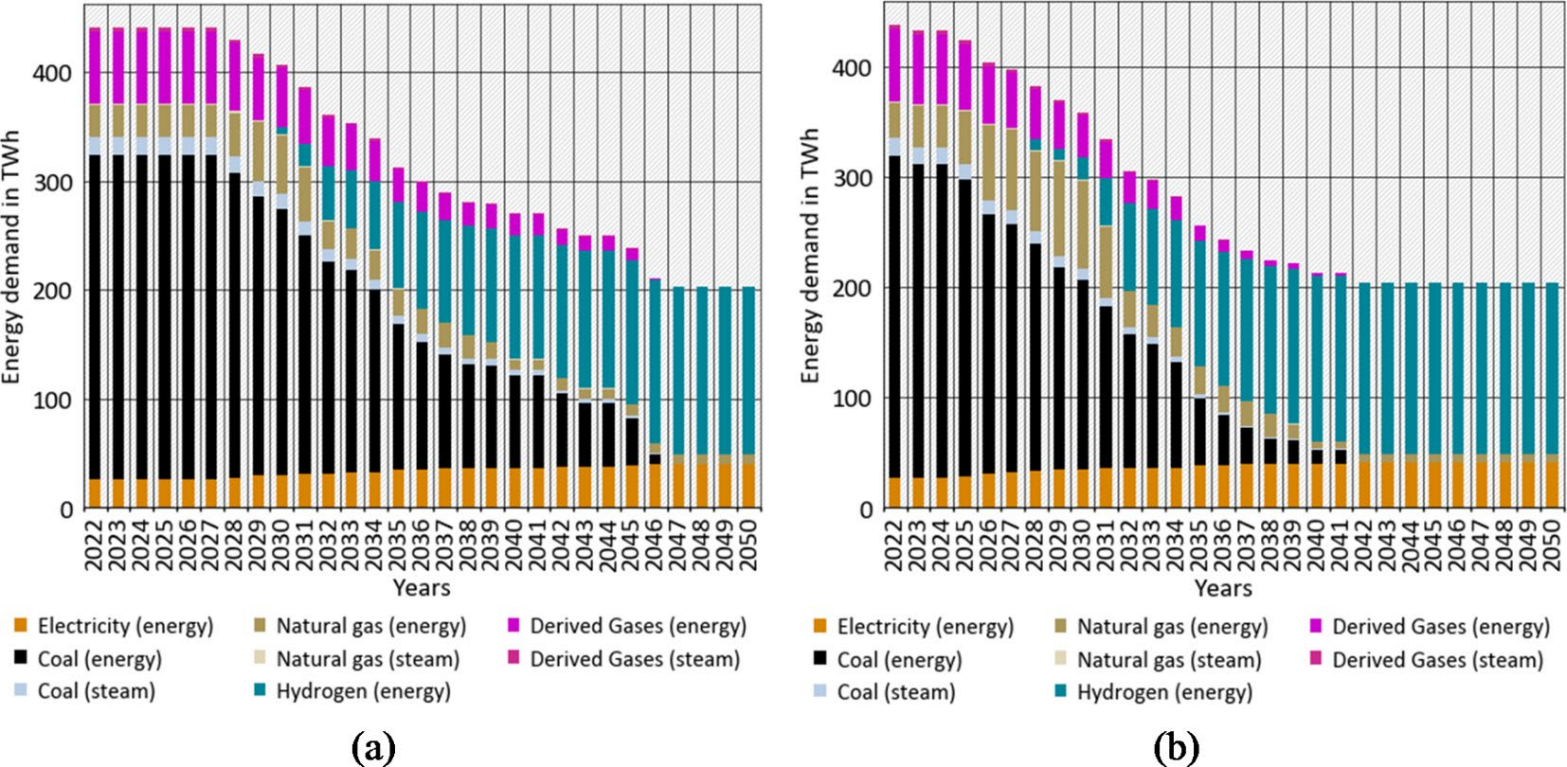
Development of the final energy demand for primary steel production in Europe without a process ban (**a**) and with forbidden reinvestments into blast furnaces (**b**).

Early reinvestments into blast furnaces for CASE 1 (Fig. 8a) lead to a high share of coal in the final energy demand until 2030. Between 2028 and 2038, a very linear decrease in coal use can be observed due to substitution with DRI units. During this substitution, natural gas-fired DRI are predominant first, and over time, natural gas will be completely replaced by hydrogen. However, there are no blast furnaces using coal in the system until 2050. In CASE 2 (Fig. 8b), the use of coal will nearly disappear until 2040. A higher natural gas demand results from early DRI investments in natural gas. In summary, the difference in the replacement of blast furnaces between both cases results in an earlier phase-out for CASE 2 (Fig. 8b).

#### Spatial development

**Fig. 9:**
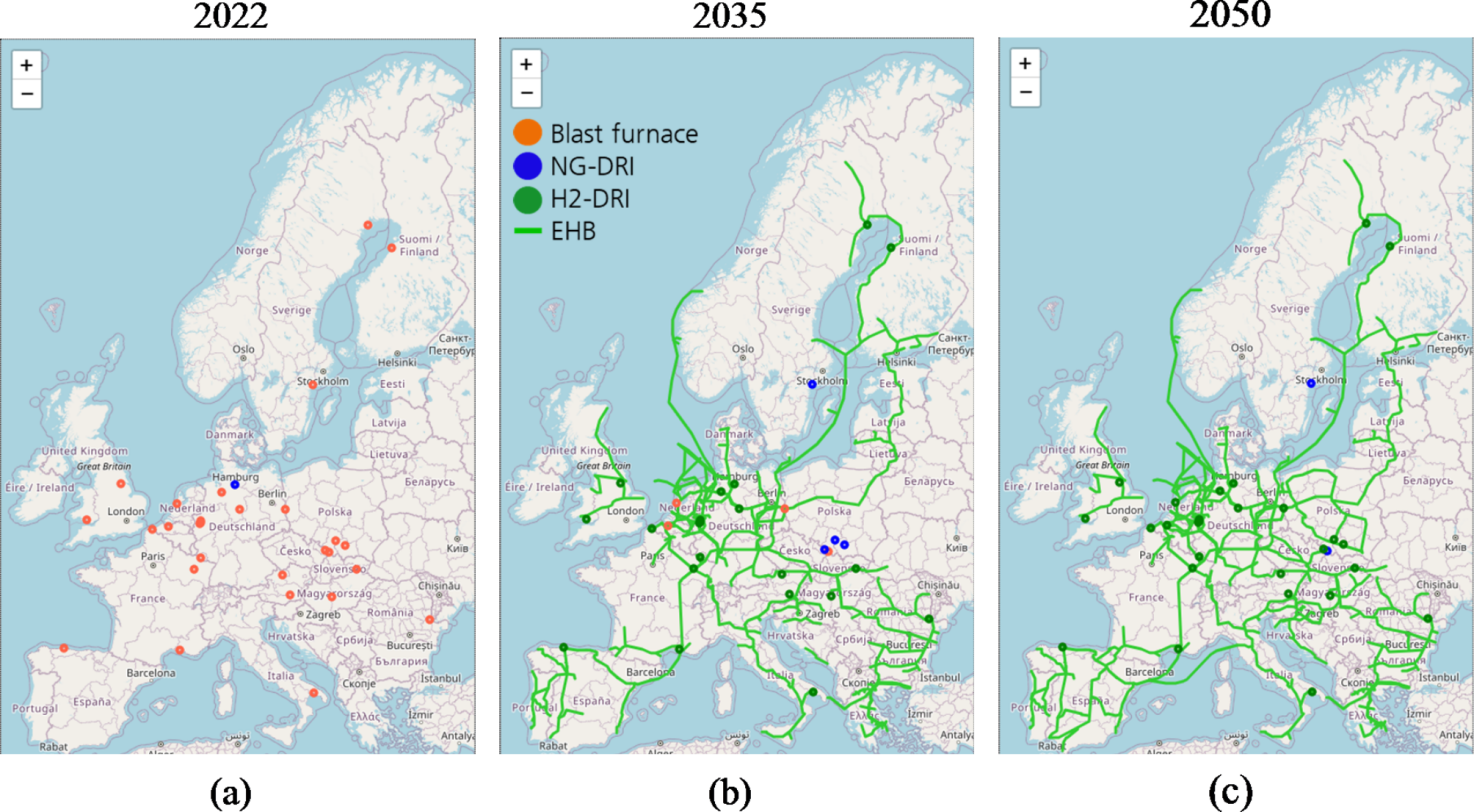
Example of the live visualisation of the model results for a transformation pathway of Europe’s primary steel production from the current state (**a**) towards 2035 (**b**) and 2050 (**c**) by recognising the uptake of the EHB.

In Fig. 9, the visualisation of the transition pathway and process switch of the conducted case study for Europe’s primary steel production is shown. Starting from today’s capital stock (a), installed blast furnaces are predominant. By 2035 (b), the hydrogen infrastructure plans according to the EHB will be nearly fully established, and individual sites will change processes towards the DRI, either by hydrogen if infrastructure connections are already established or natural gas. At the end of the simulation in 2050 (c), nearly all sites have access to hydrogen according to the plans of the EHB initiative and changed towards H_2_-DRI.

**Fig. 10:**
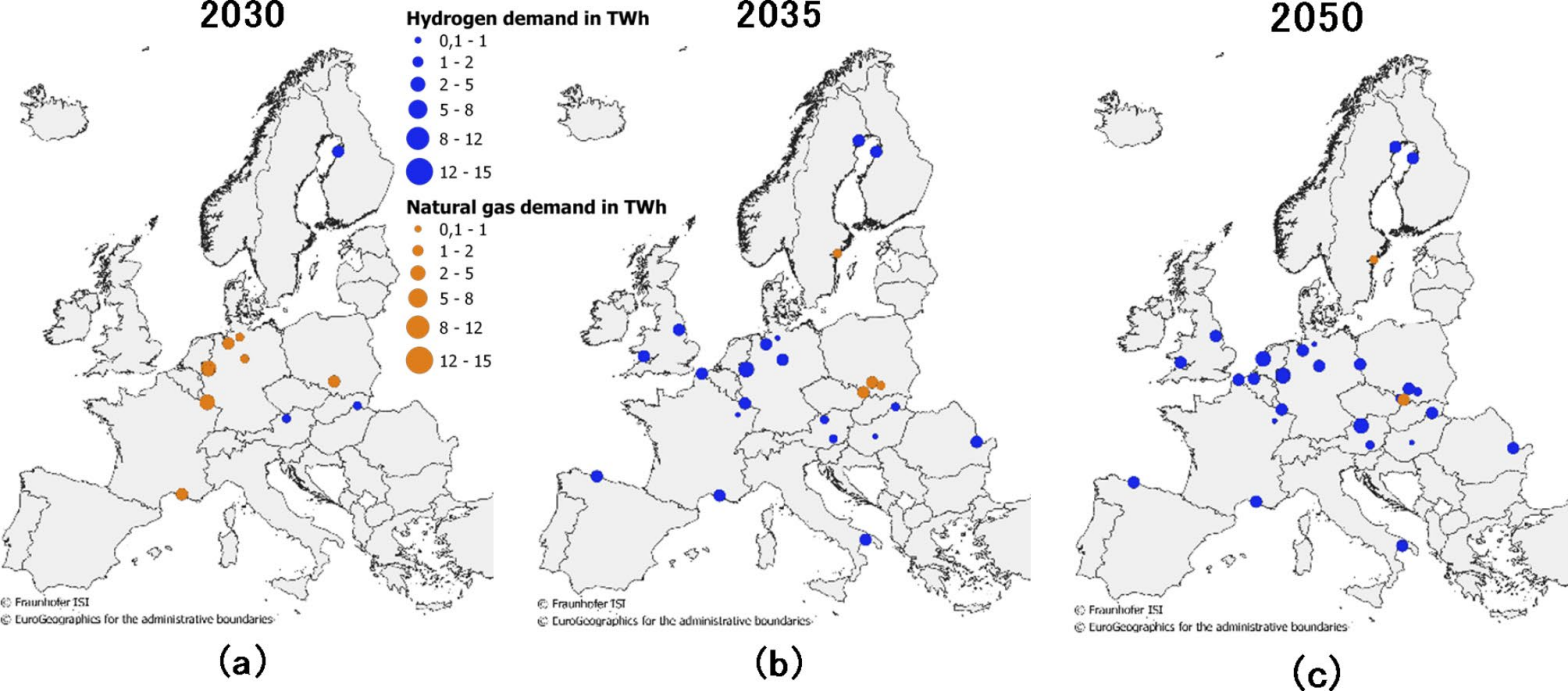
Spatial development of process diffusion in Europe’s primary steel production towards DRI fuelled with natural gas or hydrogen.

More specifically, the diffusion for DRI and its spatial resolution with a fuel switch from natural gas to hydrogen is displayed in Fig. 10. The spatial development for this case study is shown for 2030, 2035 and 2050, but the results are written at a yearly resolution. Until 2030 (Fig. 10a), investments are already being made, especially in Germany, into at least one of the operating production units per site. In central Europe, natural gas dominates the energy use in DRI units. The transitions towards 2035 (Fig. 10b) and 2050 (Fig. 10c) show increasing attractiveness according to the energy price assumptions and further investments in DRI using hydrogen. Only a few sites have no access to hydrogen infrastructure and thus use natural gas in their DRI units until 2050.

## Discussion

The presented model enables a site-specific resolution of the current plant stock of the energy-intensive industries. Based on this, scenarios can be conducted and analysed for a detailed examination of the transformation of the European industrial sector. The analysis of investment decisions in new processes and the estimation of future energy demand with the high spatial resolution of the model is valuable for strategic decisions. The model follows a simulation approach as its purpose is to simulate individual investment decisions, which allows to identify existing gaps from investment perspective^39^. Consequently, there is in the current state no optimisation that aims to calculate the optimal investment decisions for the entire system. The sum of investment decisions over the respective period allows conclusions about the spatial and temporal dynamics of the diffusion of industrial processes. The age of the respective plant and the average theoretical lifetime are the basis for individual investment decisions. Although single analyses initially started a similar approach, they did not seem to follow up on this^46^.

Nevertheless, the model has some limitations: a need for high quality data and exogenous assumptions, lack of direct interaction between the industry sites, and consideration of market mechanisms.

In general, modelling reliable investment decisions for industrial sites depends on appropriate mechanisms and data gathering. The accuracy and scope of the results depend on the quality of the used data. The Fraunhofer ISI IndustrialSiteDatabase provides detailed input data for energy-intensive industries in Europe, especially for primary steel and basic chemicals sites. The coverage of other energy-intensive industry sectors may lack details such as the age of the plants. Non-energy-intensive industries and cross-cutting processes such as steam generation are not explicitly considered in the data inputs. Depending on the process-specific input data, those cross-cutting technologies can be considered indirectly. The quality of the input data or data gaps can affect the accuracy and meaningfulness of the modelling results.

The level of detail of processes and process chains is adjustable but is subject to a uniform data set and its availability. The comparability of alternative processes for the same product may be affected if the level of detail is inconsistent for competing processes. The model allows to initialise a variable number of sub-production units and corresponding processes for each production unit. Currently, however, industrial plants are modelled as an integrated production unit due to data availability and granularity.

Another limitation is the lack of direct interaction between the industrial sites in the current model state. Although the industrial sites are indirectly connected through central constraints such as policy measures, existing infrastructure, and the availability of energy carriers, direct communication and interaction between industrial plants are currently not implemented. This limitation could impair the model’s ability to adequately represent complex dynamic interactions and interdependencies between the industry sites. For example, in the steel industry, the decision to build or not build a DRI plant at one site could influence the decision-making at another site.

A crucial and perhaps most significant influence on the results is the techno-economic parameterisation of the processes in combination with the exogenous scenario assumptions on energy carrier prices and policy measures, such as the CO_2_ price.

The mentioned limitations affect the model results in several ways. First, incomplete, or inaccurate input data could lead to distorted results, especially if important variables such as the plant age or specific technical details are missing. Secondly, dynamic effects and feedback among the industry sites are limited by the lack of direct interaction between the sites in the current status. The exogenous specification of key influencing factors such as energy carrier prices neglects possible market structures and market mechanisms. However, since the model only represents a part of the market, this limitation is difficult to avoid. Through iterations and feedback of results and parameters with energy system models that also represent other sectors, such market effects can be indirectly included.

Despite these limitations, the model provides valuable insights into specific research questions, particularly those raised in “Context and scope of the model”. This model is suitable for analysing scenarios that focus on the investment decisions of individual industrial plants and their temporal and spatial distribution. The site-specific approach supports strategic decisions by estimating when and where industrial actors will need infrastructure for new energy carriers and how energy demand and capacities might develop. Coupled with energy system models, the results obtained can provide a valuable basis for further energy system analyses, such as the need for energy infrastructures, the expansion of energy supply, or the interactions of imports and exports.

Research questions that require a comprehensive view of the entire energy system and participation in market mechanisms can be better covered by integrated energy system models, such as PyPSA^47^, TIMES^22,23^ or PRIMES^20^. These models offer a system-wide perspective, partially consider global markets, and can better represent the interactions between different sectors and energy carriers. They are also better suited for analysing and recognising long-term and intercontinental or even global developments where the specific details of individual industrial plants are less relevant.

A potential influence on the results could arise from the decisions made during model development regarding the data and methods used. The assumption of predictability of influencing factors such as energy carrier prices partially neglects possible uncertainties regarding future developments, which has an impact on potentially more conservative decisions. In addition, assumptions about economic parameters such as CO_2_ prices, investment costs, and energy prices strongly influence the model results and depends on the user. Similarly, specific technical assumptions about the efficiency and availability of new processes lead to distortions if they do not reflect real conditions.

## Conclusions and outlook

The site-specific modelling approach offers a framework that is designed to analyse the diffusion of innovative production processes in energy-intensive industries by conducting energy transition scenarios. The aim is to understand the interdependencies between the industry sector and the energy system and their impact on investment decisions of decarbonisation scenarios and to support different stakeholder groups for strategic long-term planning.

The flexibility of the framework allows for a wide range of research questions, with a particular focus on process diffusion and stock turnover, its spatial distribution and infrastructure connection. Detailed knowledge about the current age structure of industry sites enhances the accuracy of the simulations and contributes to a more comprehensive understanding of industry transition dynamics. Different policy designs impact investment decisions and affect the industry’s transition to decarbonisation. Georeferenced attributes are used to assess choices based on proximity to energy infrastructure and industry clusters, which may influence process selection. The strength of the model is the modelling of site-specific investment decisions in a detailed spatial and temporal resolution, influenced by multiple factors based on techno-economic aspects.

The open-source interface of the model contributes to the scientific community’s understanding of industrial decarbonisation allowing to customise and manipulation of key parameters, facilitating further research, scenario analyses and methodological developments.

In summary, the developed model provides valuable insights into the diffusion of industrial processes at the site level and is well suited for specific strategic analyses. Nevertheless, the context of the system analysis research area and the presented limitations should always be considered when interpreting the model results.

Future developments should focus on enhancing horizontal connections and direct communication between the industry sites to use the advantages of the agent-based framework within the model is implemented. Additional aspects may be the implementation and methodological improvement of further policies, expansion of the data basis, consideration of individual company strategies, and improving the algorithms towards learning effects and for industry site dynamics.

Although the model is established as a simulation approach, implementing individual optimisation problems depending on specific research questions within the simulation workflow offers an opportunity for future directions.

## Electronic supplementary material

Below is the link to the electronic supplementary material.

Supplementary Material 1

## Data Availability

All data and source code is available. The model and respective data are available on GitHub referring to this article: https://github.com/fraunhofer-isi/forecast-sites.

## Abbreviations

ABM: Agent-based modelling
BF: Blast furnace
BF/BOF: Blast furnace/Basic oxygen furnace
CAPEX: Capital expenditure
CO_2_: Carbon dioxide
CCUS: Carbon capture and utilisation or storage
DRI: Direct reduced iron
EHB: European hydrogen backbone
ESM: Energy system model
EU-ETS: European union emission trading system
GHG: Greenhouse gas
GJ: Gigajoule
H_2_: Hydrogen
H_2_-DRI: Direct reduced iron using hydrogen
IAM: Integrated assessment model
ID: Identifier
ISI: Institute for systems and innovation research
Mt: Megaton
NG: Natural gas
NG-DRI: Direct reduced iron using natural gas
NUTS: Nomenclature of territorial units for statistics
OPEX: Operational expenditure
SEC: Specific energy consumption
SQL: Structured query language
TWh: Terawatt hours
