# Supplementary material for: Modelling the transformation of energy-intensive industries based on site-specific investment decisions
Source: Sci Rep. 2024 Dec 18;14:30552. doi: 10.1038/s41598-024-78881-7 (PMC11655855; doi:10.1038/s41598-024-78881-7)
Supplement: Supplementary file 1 — Supplementary Material 1 [file 41598_2024_78881_MOESM1_ESM.docx]

Supplementary Material

Belonging to

Modelling the transformation of energy-intensive industries based on site-specific investment decisions

Marius Neuwirth^1,2,^*, Tobias Fleiter^1^, René Hofmann^2^

^1^ Fraunhofer Institute for Systems & Innovation Research (ISI), Breslauer Str. 48, 76139 Karlsruhe, Germany

^2^ TU Wien, Institute of Energy Systems and Thermodynamics, Getreidemarkt 9, 1060 Wien, Austria

***** Corresponding author: [Marius.Neuwirth@isi.fraunhofer.de](mailto:Marius.Neuwirth@isi.fraunhofer.de); Tel.: +49-721-6809-528 (M.N.)

Content

[Supplementary material 2](#_Toc166573646)

[Supplementary material A: Object-oriented vs. functional programming 2](#_Toc166573647)

[Supplementary material B: Filling input data: Age distribution of the capital stock 2](#_Toc166573648)

[Supplementary material C: Cumulative investments of the conducted case study 4](#_Toc166573649)

[Supplementary material D: Packages 4](#_Toc166573650)

[Supplementary material information 4](#_Toc166573651)

[Acknowledgements 4](#_Toc166573652)

[Competing interests 4](#_Toc166573653)

[Author contributions statement 5](#_Toc166573654)

[Data availability 5](#_Toc166573655)

[References supplement 6](#_Toc166573656)

# Supplementary material

## **Supplementary material A:** Object-oriented vs. functional programming

**Table 1** briefly compares the most important indicators between the two main approaches of model implementations object-oriented and functional programming based on [1].

**Table 1:** Comparison of the advantages and disadvantages of object-oriented versus functional programming

| **Indicator** | **Functional programming** | **Object-oriented programming** |
| --- | --- | --- |
| Definition | Emphasises on the evaluation of functions | Based on the conceptualisation of objects |
| Data | Uses immutable data | Uses immutable and mutable data |
| Model | Declarative programming | Imperative programming |
| Support | Supports parallel programming | Does not support parallel programming |
| Execution | No strict order of execution | Strict order of execution |
| Iteration | Recursion for iterative data | Loops for iterative data |
| Elements | Variables and functions | Objects and methods |
| Use | Few things with many operations | Many things with few operations |

In summary, the key benefit of object-oriented programming for our use case is the easier structuring of large amounts of data, which are necessary for rebuilding complex industry structures. In addition, an object-oriented programming style fits best for the requirement of implementing as an agent-based model.

## *Supplementary material* B: Filling input data: Age distribution of the capital stock

To depict diffusion with high resolution, the model requires knowledge about the age per production unit in the preceding step. **Figure 1** shows the algorithm for this approach to depict the ages of all existing production units in energy-intensive industries individually.

**Figure 1:** Algorithm for estimating missing input data on the age distribution of the capital stock

Here, we follow two parallel strands due to differences in data availability for different industry sectors and sites, as defined by equation 1.

| $plant age=\left\{ \begin{aligned} yes=1, &Directly feed into discrete reinvestment decision \\ no =0, &Add to stock model before discrete decision \end{aligned} \right.$ |  | (1) |
| --- | --- | --- |

Production units with ages from primary sources are directly fed to the reinvestment decision by applying the diffusion mechanism. In the second case, for all production units where the age is not known, the initialisation of the stock model is carried out by summing the number of production units per product and region that do not contain any information about the age. Then, all production units of the respective product and region containing information about age are summed up for building a histogram such as the exemplary for Iron and Steel in Europe in **Figure 2**. Based on the histogram of the known ages and the typical lifetimes of the processes, a distribution that fits the empirical data is chosen. Finally, specific years for the next reinvestments are determined by following this distribution and are allocated to the production units missing ages.


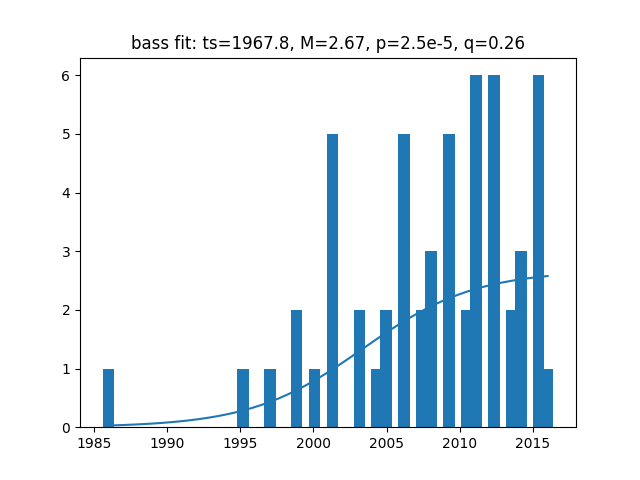


**Figure 2:** Exemplary Bass fit for the European iron and steel industry to investigate an age distribution applied to production units without exact knowledge about the age

The allocation of the age structure can be represented by various statistical distributions in diffusion theories. Here, the basis for the algorithm developed in this analysis is the Bass model [2]. As shown in **Figure 2**, a fit for the Bass model approach is to investigate the parameters needed to adapt the distribution of the Bass model to the empirical data. The key performance indicator calculated with this approach is the number of adoptions per time period, represented by the mathematical formulation of the differential equation in equation 2.

| $n\left( t \right)=\frac{dN(t)}{dt}=m\cdot\left( p+q\cdot\frac{N\left( t \right)}{m} \right)\cdot\left( 1-\frac{N\left( t \right)}{m} \right)=m\cdot p+\left( q-p \right)\cdot N(t)-\frac{q}{m}\cdot{N(t)}^{2}$ |  | (2) |
| --- | --- | --- |

$t$ time step/period

$n\left( t \right)$ number of adoptions per time step t

$N\left( t \right)$ cumulative adoptions until time period t

$p$ coefficient for early ramp-up

$q$ coefficient for the increase in adoption

$m$ market potential

The cumulative adoptions per time period $N\left( t \right)$ are given through the solution of the differential equation:

| $N(t)=m\cdot\left[ \frac{q{-p\cdot e}^{-\left( p+q \right)t}}{{q\cdot(1+e}^{-\left( p+q \right)t})} \right]$ |  | (3) |
| --- | --- | --- |

For the number of adoptions per time step, the discrete result of the differential equation yields the time-domain representation, in which the number of adoptions only depends on time and not on the cumulative adoptions:

| $n\left( t \right)=\frac{dN(t)}{dt}=\left\{ \begin{aligned} N(t), &t=1 \\ N\left( t \right)-N(t-1), &t>1 \end{aligned} \right.$ |  | (4) |
| --- | --- | --- |

The course of the diffusion curve described by the Bass model depends on the level of the three parameters to be estimated: $p$, $q$ and $m$. Here, $p$ determines the early ramp-up of the diffusion process due to external influences, while $q$ is responsible for the rise of adoption influenced by the amount of adoption thus far and an exhaustion of the market potential $m$. The market potential does not influence the course of the diffusion curve but rather the absolute level of adoption, which is given by the number of missing ages per product, production unit and region. If $p=0$, the distribution equals a logistic distribution. If $q=0$, the Bass model follows an exponential distribution.

## **Supplementary material C:** Cumulative investments of the conducted case study

Short description of the two investigated cases:

- CASE 1: **Figure 3** (a) represents the cumulative investments without applying a ban for reinvestments into the blast furnace process. As a result, in the early years blast furnaces remain the most attractive process. In 2028, the first investments into DRI units are made and fired by natural gas. Hydrogen starts to become techno-economically attractive from 2030 onwards. In this case, 17 billion euros will be invested until 2030, of which 9 billion euros will be invested into blast furnace relining. The cumulative investment sum until 2040 would result in 52 billion euros of investments. DRI investments from 2045 to 2050 represent the beginning of a second investment phase. In total, 71 billion euros of investments are made by 2050.
- CASE 2 (**Figure 3** (b)) realises a process ban for reinvestments in blast furnaces. This reflects the announcements and strategies of European steel manufacturers [3,4]. Thus, higher investments of approximately 13 billion euros are necessary in the early years until 2030, as DRI investments are more expensive than BF relining. In summary, the cumulative investments are 30 billion euros by 2030, 67 billion euros by 2040 and 77 billion euros by 2050.

| 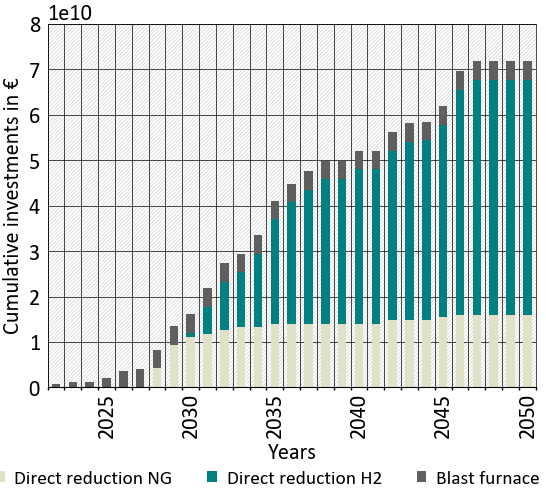  (a) | 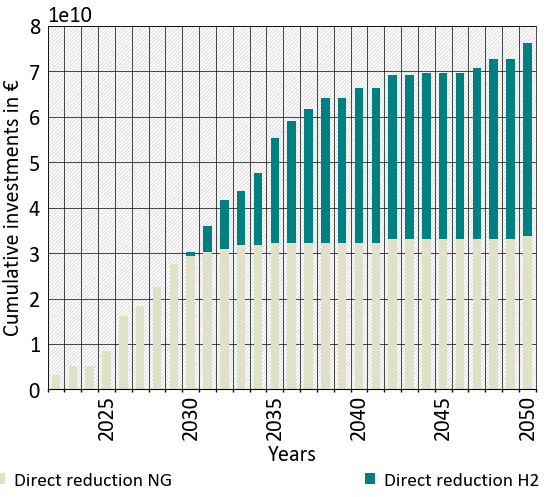  (b) |
| --- | --- |

**Figure 3:** Cumulative investments for the conducted case study for primary steel production in EU27+3 without a process ban on blast furnaces (a) and by applying a process ban (b)

## **Supplementary material D:** Packages

| **Package** | **Version*** | **Package** | **Version*** |
| --- | --- | --- | --- |
| pandas | 2.2.1 | jsonpickle | 3.0.1 |
| geopandas | 0.14.3 | autopep8 | 2.0.1 |
| matplotlib | 3.8.4 | pylint | 2.15.9 |
| fiona | 1.9.6 | pytest | 7.2.0 |
| shapely | 2.0.3 | pytest-cov | 4.0.0 |
| pyproj | 3.6.1 | coverage | 7.0.0 |
| rtree | 1.0.1 | pytest-xdist | 2.5.0 |
| mesa-geo | 0.7.1 | mock | 4.0.3 |
| scipy | 1.9.2 | mesa | 2.1.5 |

*All package version will be updated regularly. Latest version requirements are given within the repository in the corresponding requirements.txt. file.

# Supplementary material information

## Acknowledgements

This research was carried out as part of the Transience project within the European Union’s Horizon Europe research and innovation programme under the grant agreement No. 101137606.

## Competing interests

The authors declare no competing interest.

## Author contributions statement

Conceptualization, M.N., T.F. and R.H.; methodology, M.N.; software, M.N.; validation, M.N.; formal analysis, M.N.; investigation, M.N.; resources, M.N., T.F.; data curation, M.N., T.F.; writing—original draft preparation, M.N.; writing—review and editing, M.N., T.F. visualization, M.N.; supervision, T.F. and R.H.; project administration, T.F., M.N.; funding acquisition, T.F., M.N.;

All authors have read and agreed to the published version of the manuscript.

## Data availability

All data and source code is available.

During review process, the source code is available for the reviewers using the following URL: <https://owncloud.fraunhofer.de/index.php/s/2wSkGQrLpmoxLtA>

And is accessible with the following password: Sub_sci-rep_2024

After acceptance, the model and its source code will be made available on GitHub and Zenodo referring to this article: <https://github.com/M-Neuwirth/FORECAST-Sites>

References supplement

[1] Ipsita Dalai, Difference between Functional Programming and Object Oriented Programming, 2022. https://www.geeksforgeeks.org/difference-between-functional-programming-and-object-oriented-programming/ (accessed 26 December 2023).

[2] Frank M. Bass, A new product growth for model consumer durables, Management Science 1969 (1969).

[3] Eurofer, Low-CO2 emissions projects: Detailed map and pathways (2024).

[4] Eurofer, Map of Eu steel production sites (2020).
